# Supplementary material for: Identification of photoperiod-induced specific miRNAs in the adrenal glands of Sunite sheep (Ovis aries)
Source: Front Vet Sci. 2022 Jul 22;9:888207. doi: 10.3389/fvets.2022.888207 (PMC9354845; doi:10.3389/fvets.2022.888207)
Supplement: Supplementary file 1 [file Data_Sheet_1.ZIP › Supplementary file/miRDeep2 file.zip!/miRDeep2 file/Novel_Structure/SPLP42A2/Novel_709.pdf]

Provisional ID	:	NC_019460.2_9738
Score total	:	1.7
Score for star read(s)	:	-1.3
Score for read counts	:	0
Score for mfe	:	1.4
Score for randfold	:	1.6
Score for cons. seed	:	
Total read count	:	20
Mature read count	:	17
Loop read count	:	0
Star read count	:	3

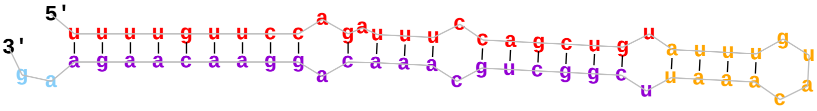

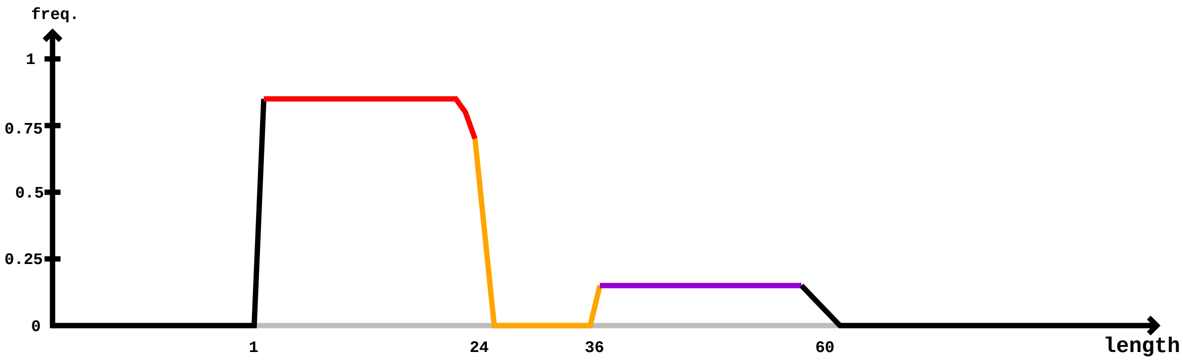


```
                     Mature                                     Star
5'- accuuuggaguuggaugacuuuuuguuccagauuuccagcuguauuuguacaaauucggcugcaaacaggaacaagaaguugucgcuuaacucaaguagauccuuggaacuga   -3'   obs
    accuuuggaguuggaugacuuuuuguuccagauuuccagcuguauuuguacaaauucggcugcaaacaggaacaagaaguugucgcuuaacucaaguagauccuuggaacuga          exp
    ..((...((((((((((((((((((((((.(.(((.((((((.((((....)))).)))))).)))).))))))))))))))))...))))))....))..............    reads    mm    sample
    ....................uuuuguuccagauuuccagcu........................................................................    1        0     seq
    ....................uuuuguuccagauuuccagcug.......................................................................    2        0     seq
    ....................uuuuguuccagauuuccagcugu......................................................................    11       0     seq
    ....................uuuuguuccagauuuccagcugua.....................................................................    3        0     seq
    .......................................................ucggcugcaaacaggaacaaga....................................    3        0     seq
```
